# Inhibition of miR-128-3p by Tongxinluo Protects Human Cardiomyocytes from Ischemia/reperfusion Injury via Upregulation of p70s6k1/p-p70s6k1

**DOI:** 10.3389/fphar.2017.00775

**Published:** 2017-10-30

**Authors:** Gui-hao Chen, Chuan-sheng Xu, Jie Zhang, Qing Li, He-he Cui, Xiang-dong Li, Li-ping Chang, Rui-jie Tang, Jun-yan Xu, Xia-qiu Tian, Pei-sen Huang, Jun Xu, Chen Jin, Yue-jin Yang

**Affiliations:** ^1^State Key Laboratory of Cardiovascular Disease, Department of Cardiology, Fuwai Hospital, National Center for Cardiovascular Diseases, Chinese Academy of Medical Sciences and Peking Union Medical College, Beijing, China; ^2^Department of Cardiac Surgery, Shandong Provincial Hospital Affiliated to Shandong University, Jinan, Shandong, China; ^3^Department of Cardiology, Beijing Friendship Hospital, Capital Medical University, Beijing, China; ^4^National Key Laboratory of Collateral Disease Research and Innovative Chinese Medicine, Shijiazhuang, China; ^5^Department of Surgical Intensive Care Unit, Beijing Anzhen Hospital, Capital Medical University, Beijing, China

**Keywords:** myocardial reperfusion injury, cardioprotection, tongxinluo, human cardiomyocytes, apoptosis, p70s6k1, miR-128-3p, translational medicine

## Abstract

**Background and Aims:** Tongxinluo (TXL) is a multifunctional traditional Chinese medicine that has been widely used to treat cardiovascular and cerebrovascular diseases. However, no studies have explored whether TXL can protect human cardiomyocytes (HCMs) from ischemia/reperfusion (I/R) injury. Reperfusion Injury Salvage Kinase (RISK) pathway activation was previously demonstrated to protect the hearts against I/R injury and it is generally activated via Akt or (and) Erk 1/2, and their common downstream protein, ribosomal protein S6 kinase (p70s6k). In addition, prior studies proved that TXL treatment of cells promoted secretion of VEGF, which could be stimulated by the increased phosphorylation of one p70s6k subtype, p70s6k1. Consequently, we hypothesized TXL could protect HCMs from I/R injury by activating p70s6k1 and investigated the underlying mechanism.

**Methods and Results:** HCMs were exposed to hypoxia (18 h) and reoxygenation (2 h) (H/R), with or without TXL pretreatment. H/R reduced mitochondrial membrane potential, increased bax/bcl-2 ratios and cytochrome C levels and induced HCM apoptosis. TXL preconditioning reversed these H/R-induced changes in a dose-dependent manner and was most effective at 400 μg/mL. The anti-apoptotic effect of TXL was abrogated by rapamycin, an inhibitor of p70s6k. However, inhibitors of Erk1/2 (U0126) or Akt (LY294002) failed to inhibit the protective effect of TXL. TXL increased p70s6k1 expression and, thus, enhanced its phosphorylation. Furthermore, transfection of cardiomyocytes with siRNA to p70s6k1 abolished the protective effects of TXL. Among the micro-RNAs (miR-145-5p, miR-128-3p and miR-497-5p) previously reported to target p70s6k1, TXL downregulated miR-128-3p in HCMs during H/R, but had no effects on miR-145-5p and miR-497-5p. An *in vivo* study confirmed the role of the p70s6k1 pathway in the infarct-sparing effect of TXL, demonstrating that TXL decreased miR-128-3p levels in the rat myocardium during I/R. Transfection of HCMs with a hsa-miR-128-3p mimic eliminated the protective effects of TXL.

**Conclusions:** The miR-128-3p/p70s6k1 signaling pathway is involved in protection by TXL against HCM apoptosis during H/R. Overexpression of p70s6k1 is, therefore, a potential new strategy for alleviating myocardial reperfusion injury.

## Introduction

Coronary heart disease is the leading cause of death worldwide. For patients undergoing an acute myocardial infarction, timely and successful myocardial reperfusion, with the implementation of thrombolytic therapy or primary percutaneous coronary intervention (PCI), is the most effective strategy for salvaging endangered cardiomyocytes and, thus, improving clinical prognosis (Anderson and Morrow, 2017). However, the process of restoring blood flow to the ischemic myocardium can, paradoxically, induce injury, through a process known as myocardial ischemia/reperfusion injury (MIRI). It was estimated that reperfusion can reduce myocardial infarct size by 40%, while a proportion of the remaining 30% infarct volume results from MIRI and, theoretically, is avoidable (Yellon and Hausenloy, 2007). Activation of the Reperfusion Injury Salvage Kinase (RISK) Pathway by pharmacological (Gao et al., 2002; Kis et al., 2003a; Gross et al., 2004; Tissier et al., 2007; Penna et al., 2012; Zhou et al., 2015) or non-pharmacological (Juhaszova et al., 2004; Tsang et al., 2004; Hausenloy et al., 2005; Zhu M. et al., 2006; Jin et al., 2008) stimulation was shown in dozens of preclinical studies to reduce the size of myocardial infarcts resulting from reperfusion injury. Therefore, pharmacological agents that activate the RISK associated kinases might have powerful cardioprotective properties. These potential target kinases include phosphatidylinositol-4,5-bisphosphate 3-kinase/protein kinase B (PI3K/Akt), extracellular signal–regulated kinase (Erk) and their downstream targets, ribosomal protein S6 kinase (p70s6k) and glycogen synthase kinase 3β (GSK 3β) (Heusch, 2015).

Tongxinluo (TXL) is a traditional Chinese medicine that was registered with the China State Food and Drug Administration (CFDA) in 1996. The major ingredients of TXL are extracted from Radix ginseng, Buthus martensi, Hirudo, Eupolyphaga seu steleophaga, Scolopendra subspinipes, Periostracum cicadae, Radix paeoniae rubra, Semen ziziphi spinosae, Lignum dalbergiae odoriferae, Lignum santali albi, and Borneolum syntheticum (Karalliedde and Kappagoda, 2009). Its active constituents include peoniflorin, ginsenoside Rg1, ginsenoside Rb1, jujuboside A, jujuboside B, isoborneol and borneol (Chen et al., 2009). Previous studies demonstrated protective effects of TXL in a number of diseases, such as angina pectoris (Jia and Leung, 2015), atherosclerosis (Wu et al., 2015), pulmonary hypertension (Wang Y. et al., 2016), hypertension (Wang J. et al., 2014) and cerebral ischemic infarction (Cai et al., 2016). Although, our previous studies proved that TXL could attenuate myocardial reperfusion injury (MIRI) in an endothelial nitric oxide synthase (eNOS)-related pathway (Cheng et al., 2009; Li et al., 2010), whether TXL can protect human cardiomyocytes (HCMs) from reperfusion injury remains unknown. Consequently, our study aimed to investigate whether TXL would exert protective effects on HCMs during reperfusion and, if so, to identify the underlying mechanisms.

## Materials and methods

### Preparation of TXL solution

A solution of TXL ultrafine powder (Lot Number: 071201; Shijiazhuang Yiling Pharmaceutical Co., Shijiazhuang, China) was prepared as previously described, with minor modifications (Liang et al., 2011). Briefly, after the powder was dissolved in serum-free Dulbecco's modified Eagle's medium (DMEM; Life Technologies, Grand Island, NY, USA), the suspension was sonicated for 30 min and then centrifuged it at 2,500 rpm for 15 min. Sterile TXL solution was obtained by filtering the supernatant through a 0.22-μm filter. The precipitate was then dried, enabling precise weighing of the dissolved TXL powder. The solution was adjusted to a final concentration of 2,000 μg/mL by adding DMEM and was then stored at 4° or −20°C until use.

### Cell viability assay

To assess cell viability, 4 × 10^3^ HCMs were seeded per well in a 96-well plate. To determine the toxicity of TXL in HCMs, various groups of cells were pretreated with TXL at different concentrations (0, 100, 200, 400, 800, and 1,200 μg/mL) for 24 h, under normal culture conditions, before assessing cell viability. To assay protective effects of TXL on HCMs, cell viability assays were performed after H/R. Each group contained triplicate wells in every independent experiment. HCM viability was determined using WST tetrazolium salt (CCK-8, Dojindo) according to the manufacturer's instructions. Briefly, CCK-8 reagent (10 μl) was added to each well and the plates were incubated at 37°C for 3 h. Absorbances at 450 nm were then determined with a microplate reader.

### Animals

Male Sprague Dawley rats (220–250 g) were used in this study. Animal experiments were performed in accordance with the “Guide for the Care and Use of Laboratory Animals” issued by the US National Institutes of Health (Bethesda, MD, USA, NIH Publication No. 85-23, revised 1996) and the “Regulation to the Care and Use of Experimental Animals” of the Beijing Council on Animal Care (1996). The study protocol was approved by the Care of Experimental Animals Committee of Fuwai Hospital.

### Cell culture and treatments

HCMs isolated from the ventricles of the adult heart were from PromoCell (Heidelberg, Germany). Cells were grown in a monolayer to 80% confluence and then subcultured using Ready-to-Use Myocyte Growth Medium (PromoCell). Experiments with HCMs were performed at passages three to seven. Cells were washed with phosphate buffered saline (PBS) and exposed to the various treatments in serum-free DMEM for 30 min prior to hypoxia. HCMs were then incubated in an airtight and hypoxic GENbox jar fitted with a catalyst (BioMérieux, Marcy l'Etoile, France) to scavenge free oxygen, inducing 18 h hypoxia, as previously described (Chen J. et al., 2008) and were then moved to normal conditions for 2 h reoxygenation. An anaerobic indicator dye (BioMérieux) was used to assess the oxygen tension of the medium, which enabled confirmation of successful establishment of the *in vitro* H/R model. U0126 was used as a Mek/Erk inhibitor, LY294002 as an Akt inhibitor and rapamycin as a p70s6k inhibitor, with treatments were performed as previously described. U0126 was administered at 10 μM (Vicencio et al., 2015), LY294002 at 10 μM (Vicencio et al., 2015), and rapamycin at 10 nM (Qiu et al., 2004) in experiments to further investigate the mechanisms underlying the protective effects of TXL on HCMs.

### Assessment of morphological changes

Cell nuclear condensation and fragmentation were assessed in cells stained with the chromatin dye Hoechst 33,342 (Beyotime, China), as previously described (Zhu W. et al., 2006). Briefly, cells were fixed with 4% paraformaldehyde for 30 min and then exposed to 5 mg/mL Hoechst 33,342 for 30 min, then washed twice with PBS. Finally, stained cells were washed twice with PBS at room temperature and observed under a fluorescence microscope (Leica, Germany). Cells with fragmented and condensed apoptotic nuclei were considered to have undergone apoptosis.

### Measurement of mitochondrial membrane potential

Mitochondrial membrane potential (MMP) was assessed using the 5,5′,6,6′-Tetrachloro-1,1′,3,3′-tetraethyl-imidacarbocyanine iodide (JC-1) assay (Beyotime) following the manufacturer's instructions. In brief, HCMs cultured in 6-well plates were harvested after indicated treatments, suspended in the mixture (0.5 mL complete medium and 0.5 mL JC-1 staining solution), and then incubated at 37°C in for 20 min. Eventually, the cells were resuspended in 300 μl staining buffer and analyzed by flow cytometry (FACSAria 2, Becton-Dickinson) after being rinsed twice with ice-cold JC-1 staining buffer. The MMP of each sample were expressed as the ratio of red fluorescence intensity over green fluorescence intensity. And mitochondrial depolarization is indicated by a decrease in the red/green fluorescence intensity ratio.

### EdU assay

The effects of TXL on the proliferation of HCMs were determined by the EdU incorporation assay using the EdU assay kit (Ribobio, China) according to the manufacturer's instructions. Briefly, HCMs were cultured in 6-well plates and were incubated with TXL at different concentrations and 20 μM of EdU during H/R. Then cells were collected, washed with PBS for one time, fixed with 4% paraformaldehyde for 15 min at room temperature and treated with 0.5% Triton X-100 for 20 min at room temperature for permeabilization. After being rinsed twice with PBS, HCMs were incubated with 1 × Apollo® 488 reaction cocktail (300 μl/well) for 10 min. Then HCMs were resuspended in 300 μl PBS and analyzed by flow cytometry (FACSAria 2, Becton-Dickinson) after being washed twice with 0.5% Triton X-100. HCMs stained with Apollo® 488 dye were EdU-positive and considered to be newborn cells.

### Determination of apoptosis by flow cytometry

Cell apoptosis was assessed using the Annexin V-FITC/PI Kit (Becton, Dickinson and Company, USA), following the manufacturer's instructions. Briefly, HCMs, after experimental treatments, were collected and resuspended in 100 μl 1 × binding buffer. The cell suspension was incubated for 15 min at room temperature in the absence of light after addition of annexin V (5 μl) and propidium iodide (PI) (5 μl) solutions. Next, 400 μl 1 × binding buffer was added and HCMs were harvested and analyzed using the FACS Calibur System (Becton-Dickinson). Viable HCMs were defined as annexin V^−^/propidium iodide (PI)^−^, early apoptotic HCMs as annexin V^+^/PI^−^ and late apoptotic HCMs and necrotic HCMs as annexin V^+^/PI^+^. The proportion of apoptotic HCMs was calculated after adding together early and late apoptotic cells.

### Establishment of the *in vivo* myocardial I/R injury model

Male Sprague Dawley rats were anesthetized with sodium pentobarbital (50 mg/kg, intraperitoneally) before endotracheal intubation. I/R was induced by ligating the left anterior descending artery (LAD) for 45 min, followed by loosening the ligature for 180 min, as described previously (Kang et al., 2014). Rats were randomized to four groups: Sham group, in which LAD was encircled by a suture but not occluded; I/R group, in which rats were administered saline by gavage 1 h prior to I/R; I/R + TXL group, in which rats were administered TXL dissolved in saline (0.4 g/kg, an equivalent dose to that used clinically in humans) by gavage 1 h prior to I/R; and I/R + TXL + Rapamycin group, in which the rats were administered rapamycin (0.25 mg/kg) intravenously 30 min prior to I/R, as described previously (Wagner et al., 2010) in addition to receiving the same TXL treatment as the TXL group.

### Tunel assay

Cardiac tissues were fixed in neutral 10% formalin for 24 h, embedded in paraffin, and then cut into 5 μm thick slices. After deparaffinization and rehydration, apoptotic cardiomyocytes were detected in the myocardium using a terminal dUTP nick end-labeling (TUNEL) assay, as previously described (Chen et al., 2017). TUNEL staining was conducted with the *in situ* Cell Death Detection kit (Roche, Indianapolis, IN, USA). Cardiac slices were also stained for nuclei with 4′, 6-diamidino-2-phenylindole (DAPI) (Invitrogen, CA, USA). Finally, images were acquired with a Leica (SP8) confocal microscopy system at 400 × magnification and the average of the ratios of TUNEL-positive to total nuclei from five representative microscopic fields, obtained from the midventricular section of each heart, was calculated.

### Infarct size measurement

Infarct sizes were measured in myocardial tissue as previously described, with a minor modification (Ge et al., 2015). The suture encircling the LAD was re-tied after reperfusion, and 1 mL 2% Evans Blue dye was injected into the thoracic aorta to distinguish the ischemic area (area-at-risk, AAR) from the non-ischemic area (Evans blue perfused region). The heart was immediately harvested, once the dye was uniformly distributed, and then stored frozen at −80°C overnight. Frozen ventricles were sliced into 5 or 6 sections, which were incubated in 1% 2, 3, 5-triphenyltetrazolium chloride (TTC, Amresco) for 15 min at 37°C to stain ischemic but viable tissue (red) and to visualize the infarcted area (pale white, IA). The IA, AAR, and total cross-sectional heart area (TA) were measured using ImageJ software (National Institutes of Health). The infarct size and ischemic area were expressed as a percentage of AAR (IA/AAR) and TA (AAR/TA), respectively.

### Real-time RT-PCR

Total RNA from HCMs or heart tissue samples was extracted using Trizol reagent, according to the manufacturer's protocol (Invitrogen). The p70s6k1 and GAPDH mRNAs were reverse transcribed to cDNA using an iScript cDNA Synthesis Kit (Bio-Rad, Hercules, CA, USA) and then quantified by quantitative real-time RT-PCR with SYBR Green PCR Master Mix. MicroRNAs (miR-497-5p, miR-145-5p, and miR-128-3p) were reverse transcribed using miScript II RT Kit (Qiagen, Valencia, CA, USA) and then quantified by quantitative real-time RT-PCR using the miScript SYBR green PCR kit (Qiagen). The qRT-PCR was performed on an ABI 7500 thermocycler (Applied Biosystems) for 40 cycles. The following PCR primers were used: GAPDH forward, 5′-GAAGGTGAAGGTCGGAGTCA-3′; reverse, 5′-GGAAGATGGTGATGGGATTTC-3′; p70s6k1, forward, 5′-ACTTCTGGCTCGAAAGGTGG-3′; reverse, 5′-TTGAGTCATCTGGGCTGTCG-3′. Specific primers for miR-497-5p, miR-145-5p, miR-128-3p, and U6 were obtained from Qiagen. The p70s6k1 mRNA was quantified with the 2^(−ΔΔCT)^ relative quantification method, using GAPDH as an internal control. The miR-497-5p, miR-145-5p, and miR-128-3p levels were determined with the 2^(−ΔΔCT)^ relative quantification method, using U6 as an internal control.

### Western blotting

Proteins were extracted from HCMs or heart tissue samples. Protein concentrations were measured with the BCA assay (Beyotime, China). To evaluate protein levels in these samples, lysate samples containing 25 mg protein were separated on NuPage 4–12% Bis-Tris Gels (Novex, Life Technologies) and protein bands were transferred to nitrocellulose membranes using a dry electroblotting apparatus (Invitrogen). Then the membranes were blocked with 5% nonfat dry milk. Membranes were then incubated with primary antibodies overnight at 4°C. The primary antibodies used included ERK1/2 (1:1,000), Phospho-ERK1/2 (Thr202/Tyr204) (1:1,000), Akt (1:1,000), and Phospho-Akt (Ser473) (1:1,000), all from Cell Signaling Technology. Other primary antibodies used were p70s6k1(1:5,000), Phospho-p70s6k1 (Thr389) (1:250), Bcl-2 (1:5,000), Bax (1:5,000), and CytoC (1:5,000), all from Abcam. After incubation with primary antibodies, membranes were washed three times and incubated with appropriate secondary antibodies (1:5,000; Zhongshanjinqiao, China) the next day. After washing membranes three times, the stained bands were visualized with the Chemiluminescence Detection Kit (Pierce). Target protein signals were normalized to those of the loading control GAPDH (primary antibody at 1:5,000; Abcam, Cambridge, UK). Densitometry analysis was performed with ImageJ software.

### Cell transfection

HCMs were seeded and cultured in complete medium in 6-well plates. When HCMs reached 70–80% confluence, small interfering RNA (siRNA) oligonucleotides against p70s6k1 (Santa Cruz Biotechnology), nonspecific control siRNA oligonucleotide (Santa Cruz Biotechnology), mimics of miR-128-3p (Thermo Fisher Scientific, Inc., Waltham, MA, USA) or the microRNA negative control (Thermo Fisher Scientific, Inc.) were transfected into HCMs using Lipofectamine™ 2000 (Thermo Fisher Scientific, Inc.) in Opti-MEM® Reduced Serum Medium (Thermo Fisher Scientifc, Inc.). The proprietary sequences of siRNAs and microRNAs were not divulged by the companies.

### Statistical analysis

Quantitative data are presented as means ± standard error of the mean (SEM). One-way analysis of variance (ANOVA) was followed by post-hoc analysis by Tukey's test for multiple comparisons, using GraphPad Prism 5.01. Significance was established at the *P* < 0.05 level.

## Results

### TXL inhibited H/R-induced death of HCMs in a dose-dependent manner

**Figure 1:**
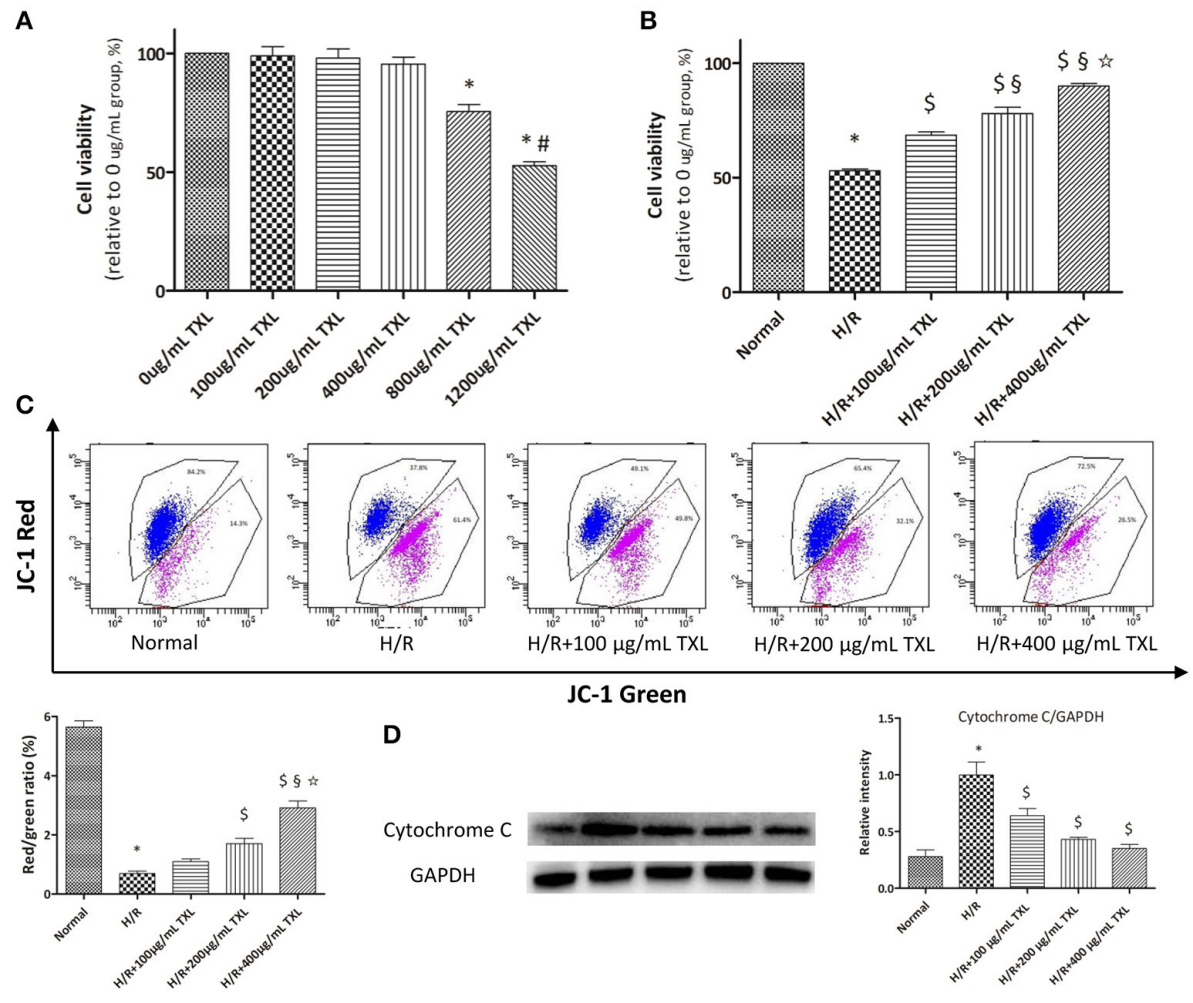
TXL dose-dependently reduced the H/R induced death of HCMs. **(A)** CCK-8 assay evaluated the toxicity of TXL on HCMs under normal condition (*n* = 6 in each group, the cells incubated with medium without TXL were considered 100% viable). **(B)** CCK-8 assay evaluated the effects of TXL at different concentrations on HCMs during H/R (*n* = 4 in each group, the cells without treatment were considered 100% viable). **(C)** JC-1 assay evaluated the effects of TXL at different concentrations on the mitochondrial membrane potential of HCMs during H/R (*n* = 3 in each group). **(D)** Western blot analysis of cytochrome C (*n* = 3 in each group) ^*^P < 0.05 vs. Normal; ^#^*P* < 0.05 vs. 800 μg/mL TXL; ^$^*P* < 0.05 vs. H/R; ^§^*P* < 0.05 vs. H/R+100 μg/mL TXL; ✩*P* < 0.05 vs. H/R+200 μg/mL TXL.

WST-8 is a reagent dissolved in the working solution of CCK-8 kit. It can be reduced to soluble formazan by dehydrogenase in mitochondria and has little toxicity to cells. In other words, the more the soluble formazan is generated within the fixed time, the more the viable cells exist in a 96-well plate. Therefore, WST-8 was used to count the number of viable cells in our experiments. Results (Figure 1A) of the CCK-8 assay showed that, compared with the 0 μg/mL group, the 800 and 1,200 μg/mL TXL groups had decreased cell viability, to 75.49 ± 3.00% (*P* < 0.05) and 52.63 ± 1.80% (*P* < 0.05), respectively, of values under normal conditions. However, 100, 200, and 400 μg/mL TXL showed no detectable toxicity. Thus, we chose 100, 200, and 400 μg/mL for subsequent experiments.

As shown by the CCK-8 assay results, cell viability was decreased to 52.93 ± 0.86% (*p* < 0.05) after exposure to H/R, indicating that we had established a cell-based model of I/R (Figure 1B). This loss of viability was prevented by TXL pretreatment, in a dose-dependent manner, with the greatest effectiveness at 400 μg/mL (89.91 ± 1.03% vs. 52.93 ± 0.86% in the H/R group, *P* < 0.05).

### TXL pretreatment protected the mitochondria of HCMs from H/R-induced injury

It is well acknowledged that the mPTP not only is central in mitochondrial damage and cell death during I/R, but is also a converging target of cardioprotective signaling (Heusch, 2015). As mPTP opening would lead to depolarization of the inner MMP, we detected pore opening using the JC-1 assay to investigate whether TXL would inhibit mPTP opening. JC-1 existed as an aggregated form (red fluorescence) in the matrix of mitochondria with the normal MMP, and it was converted to the monomeric form (green fluorescence) with the loss of MMP. Therefore, decrease in the red/green fluorescence intensity ratio could indicate the reduction of MMP. Compared with the normal cells, HCMs exposed to H/R exhibited low MMP (5.64 ± 0.21 vs. 0.69 ± 0.07 in the H/R group, *P* < 0.05). However, pretreatment of HCMs with TXL (100, 200, or 400 μg/mL) increased the red/green ratio after H/R in a dose-dependent manner (Figure 1C). This was consistent with the trend shown by the CCK-8 assay.

As mPTP opening was reported to result in release of the pro-apoptotic protein cytochrome C, we used western blotting to determine whether TXL pretreatment would affect this process. Groups treated with TXL had lower cytochrome C levels than the H/R group, especially at 400 μg/mL TXL (0.35 ± 0.04 vs. 1.00 ± 0.11 in the H/R group, *P* < 0.05) (Figure 1D).

### TXL decreased H/R-induced HCM apoptosis in a dose-dependent manner

**Figure 2:**
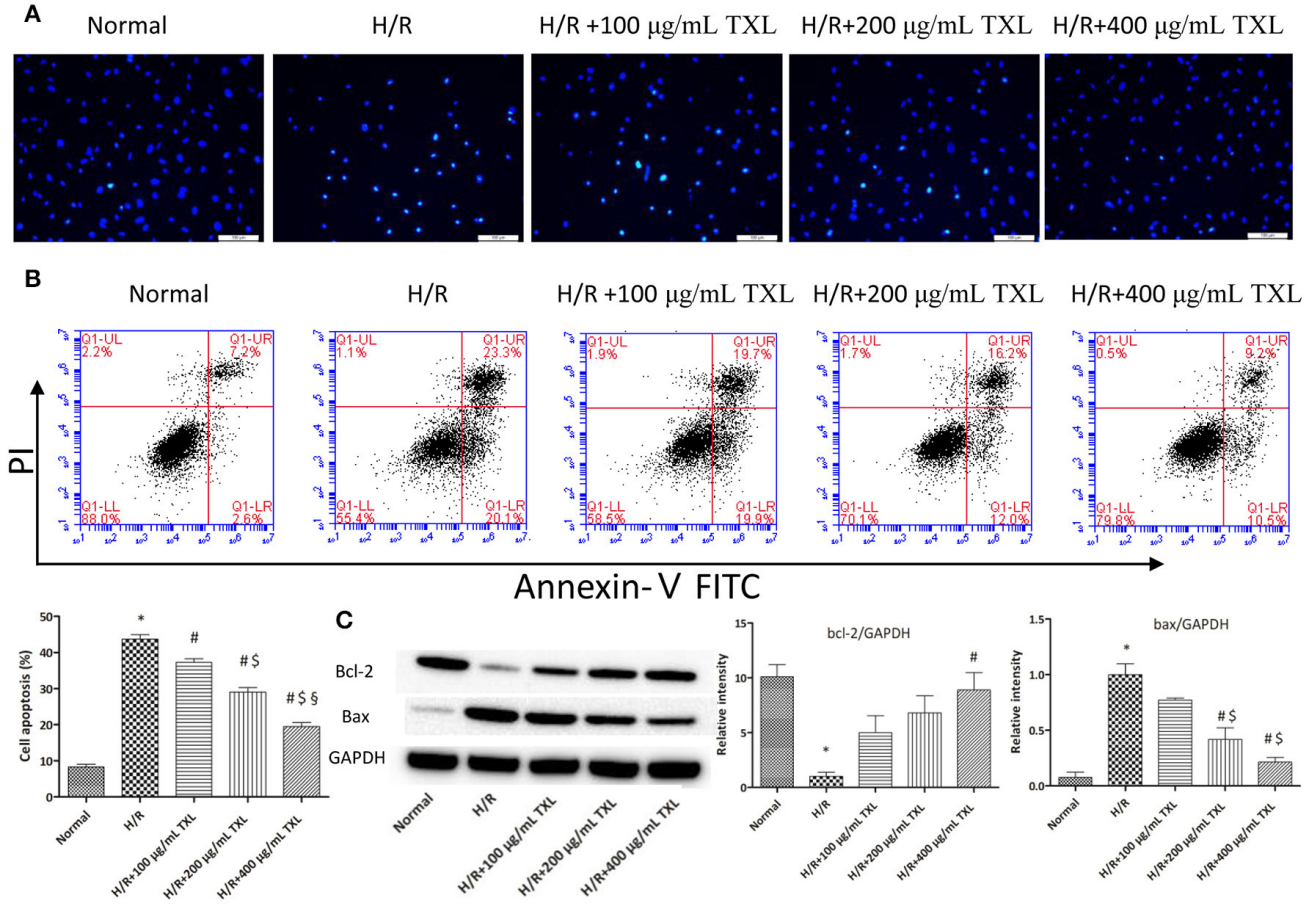
TXL reduced the H/R induced death of HCMs by inhibiting apoptosis. **(A)** Representative images on the apoptosis of HCMs obtained by Hochest staining (magnification × 200). **(B)** Representative images and quantitative data on the apoptosis of HCMs detected by flow cytometry after staining with Annexin V and propidium iodide (PI) (*n* = 4 in each group). **(C)** Western blot analysis of Bcl-2 and Bax (*n* = 3 in each group); ^*^P < 0.05 vs. Normal; ^#^*P* < 0.05 vs. H/R; ^$^*P* < 0.05 vs. H/R+100 μg/mL; ^§^ *P* < 0.05 vs. H/R+200 μg/mL.

We previously showed that TXL decreased apoptosis in mesenchymal stem cells under hypoxia and serum deprivation conditions (Li et al., 2014). Therefore, we hypothesized that TXL would alleviate H/R-induced injury in HCMs by inhibiting their apoptosis. Consequently, HCMs without TXL or with TXL at various concentrations (100, 200, or 400 μg/mL) were analyzed, by morphology and flow cytometry, to detect the anti-apoptotic effects of TXL in HCMs under H/R conditions. Hoechst 33342 is a kind of blue fluorescent dye. As it is cell-permeable and can bind to DNA in live or fixed cells, Hoechst 33342 is generally used to assess nuclear morphological changes of cells after stimulation. As shown in Figure 2A, compared with cells in the normal group, cells exposed to H/R had shrunken and condensed nuclei. In contrast, TXL, in a dose-dependent manner, prevented the changes in nuclei induced by H/R, with 400 μg/mL being the most effective concentration.

The apoptotic cells were then quantified by flow cytometry, after staining with annexin V and PI. Annexin V-FITC detects cells in early apoptosis by staining phosphatidylserine translocated to the external surface of the plasma membrane, whereas PI mainly detects late apoptotic and necrotic cells (Vermes et al., 2000; Fimognari et al., 2001). As shown in Figure 2B, flow cytometry results indicated that H/R significantly increased the rate of apoptotic HCMs, compared with in the normal group (43.70 ± 1.28% vs. 8.25 ± 0.77%, respectively, *P* < 0.05). On the contrary, TXL treatment decreased H/R-induced HCM apoptosis, in a concentration-dependent manner. TXL was anti-apoptotic at 100 μg/mL (37.23 ± 1.01% vs. 43.70 ± 1.28% in the H/R group, *P* < 0.05) and reached its peak at 400 μg/mL (19.50 ± 1.08% vs. 43.70 ± 1.28% in the H/R group, *P* < 0.05). Consequently, 400 μg/mL TXL was used for subsequent experiments.

The balance of the pro-apoptotic protein Bax and anti-apoptotic protein Bcl-2 is significant for regulating mitochondrial integrity and cell survival (Cory et al., 2003; Gustafsson and Gottlieb, 2008). To explore whether the anti-apoptotic effects of TXL were associated with Bcl-2 family proteins, expression of Bax and Bcl-2 in HCMs was determined by western blotting (Figure 2C). Compared with the H/R group, the group treated with 400 μg/mL TXL had significantly lower Bax expression (0.21 ± 0.04 vs. 1.00 ± 0.10 in the H/R group, *P* < 0.05) and also had clearly increased Bcl-2 expression (8.90 ± 1.60 vs. 1.00 ± 0.38 in the H/R group, *P* < 0.05).

### TXL activated the risk pathway by upregulating p70s6k1 and increasing p-p70s6k1 *in vitro*

**Figure 3:**
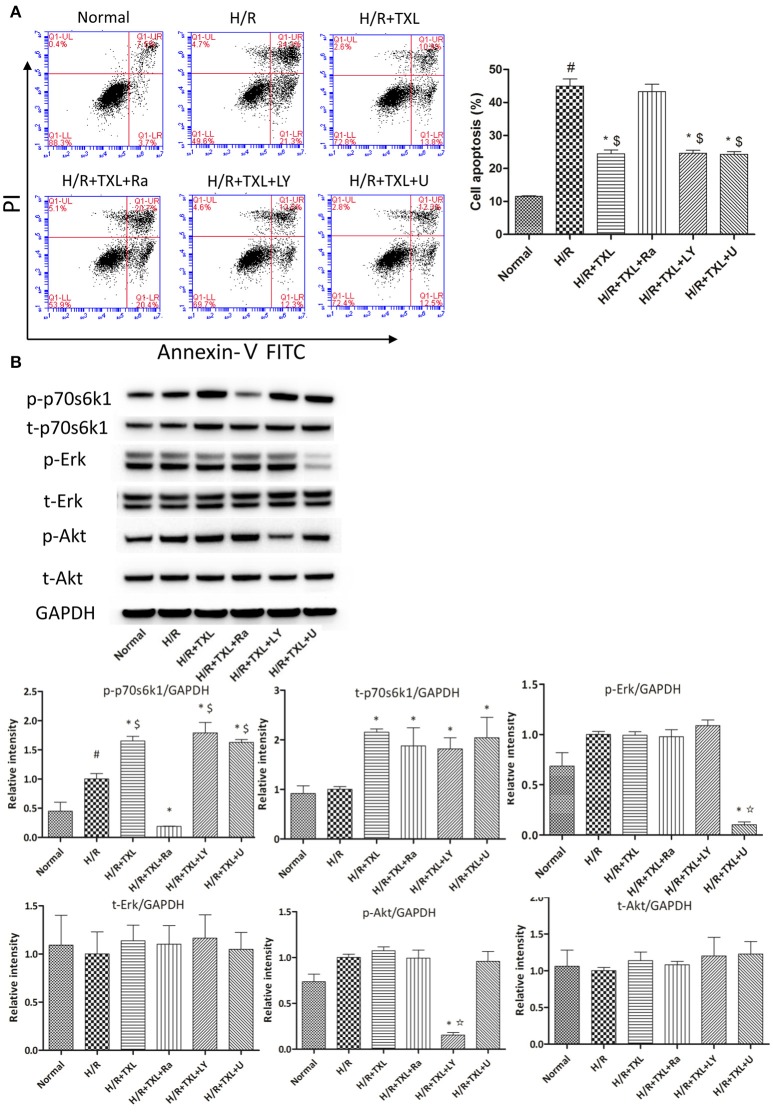
p70s6k1-related pathway mediated the anti-apoptotic effect of TXL on HCMs during H/R. **(A)** The inhibitor of p70s6k1 (rapamycin), but not that of Akt (LY294002) or Erk (U0126), abolished the anti-apoptotic effect of TXL, detected by flow cytometry (*n* = 3 in each group; rapamycin. LY294002 and U0126 alone did not increase the death of HCMs, data not shown). **(B)** Rapamycin inhibited the increased phosphorylation of p70s6k1 induced by TXL in HCMs (*n* = 4 in each group). ^*^P < 0.05 vs. H/R; ^#^*P* < 0.05 vs. normal; ^$^*P* < 0.05 vs. H/R+TXL+Ra; ✩*P* < 0.05 vs. H/R+TXL; Ra, rapamycin; LY, LY294002; U, U0126.

After demonstrating the anti-apoptotic effects of TXL in HCMs under H/R conditions, we investigated the underlying mechanisms. RISK pathway activation was reported to protect the heart against reperfusion injury (Hausenloy and Yellon, 2004; Heusch, 2015) and it is generally activated via either Akt or Erk 1/2. Thus, flow cytometry and western blotting were used to determine the roles of Akt and Erk 1/2 in the anti-apoptotic effects of TXL. Previous studies demonstrated that TXL treatment of cells promoted secretion of vascular endothelial growth factor (VEGF) (Wang et al., 2008; Hu et al., 2011). This effect was confirmed in our protein antibody arrays. Compared with the H/R group, TXL treatment groups had significantly higher levels of VEGF release by cardiac microvascular endothelial cells under H/R condition (Cui et al., 2016). Because phosphorylation of p70s6k1, a common downstream protein of Akt and Erk1/2 in the RISK pathway, was reported to stimulate VEGF expression (Skinner et al., 2004; Fang et al., 2005; Zhou et al., 2007), we also explored whether p70s6k1 mediated the protective effects of TXL. Compared with the H/R group, TXL treated groups had increased p70s6k1 expression (2.15 ± 0.04 vs. 1.00 ± 0.03 in H/R group, *P* < 0.05). Thus, TXL promoted phosphorylation of p70s6k1 at Thr389 (1.65 ± 0.04 vs. 1.00 ± 0.09 in the H/R group, *P* < 0.05), but did not affect the upstream proteins (p-Akt/Akt and p-Erk1/2/Erk1/2) of p70s6k1 in the RISK pathway (Figure 3B).

To determine whether the protective effects of TXL on HCMs were dependent on p70s6k1, p70s6k1 activation was inhibited with rapamycin, an mTOR/p70s6k inhibitor. As shown by western blotting, in the group treated with 400 μg/mL TXL, p-p70s6k1 levels were suppressed by rapamycin (0.19 ± 0.01 vs. 1.65 ± 0.04 without rapamycin, *P* < 0.05; (Figure 3B). Furthermore, rapamycin abrogated the anti-apoptotic effects of TXL on HCMs during H/R, a finding also supported by the flow cytometry results (43.3 ± 2.18% vs. 24.5 ± 1.13% in 400 μg/mL TXL-treated group, *P* < 0.05; (Figure 3A).

**Figure 4:**
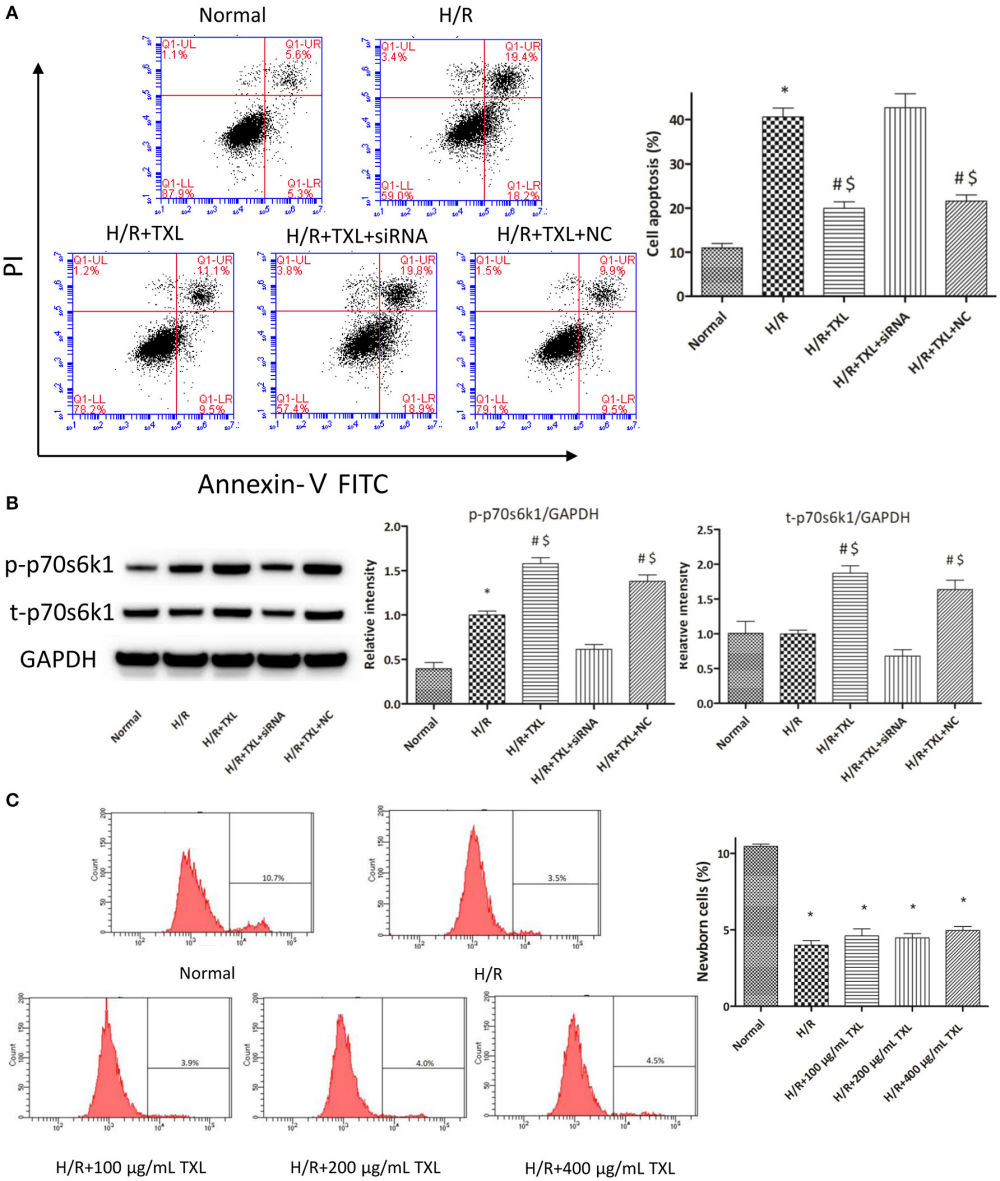
TXL activated the RISK pathway by increasing the protein level of p70s6k1 during H/R. **(A)** The siRNA to p70s6k1 abolished the anti-apoptotic effect of TXL, detected by flow cytometry (*n* = 3 in each group; siRNA to p70s6k1 alone did not increase the death of HCMs, data not shown). **(B)** The siRNA to p70s6k1 decreased the protein level of p70s6k1 in HCMs and thus inhibited the increased phosphorylation of p70s6k1 induced by TXL in HCMs (*n* = 4 in each group). **(C)** TXL did not promote the proliferation of HCMs in glucose/serum-free medium during H/R, indicated by the EdU-positive cells stained with Apollo 488 dye (*n* = 3 in each group). ^*^P < 0.05 vs. normal; ^#^*P* < 0.05 vs. H/R; ^$^*P* < 0.05 vs. H/R+TXL+siRNA; NC: siRNA negative control.

As rapamycin inhibited p70s6k1 activation via mTOR, rather than directly, and specifically affected p70s6k1 phosphorylation, we used siRNA against p70s6k1 to confirm the role of p70s6k1 in the beneficial effects of TXL. Transfection with siRNA against p70s6k1 significantly downregulated p70s6k1 expression (0.68 ± 0.09 vs. 1.87 ± 0.11 in the 400 μg/mL TXL-treated group, *P* < 0.05; Figure 4B) and, therefore, decreased p70s6k1 phosphorylation (0.62 ± 0.05 vs. 1.58 ± 0.07 in the 400 μg/mL TXL-treated group, *P* < 0.05; Figure 4B). The protective effects of TXL on HCMs during H/R were abrogated by siRNA, as evidenced by the increase in apoptotic cells detected by flow cytometry (19.90 ± 1.46% vs. 42.73 ± 3.18% in the H/R+TXL+siRNA group, *P* < 0.05; Figure 4A).

Although, the results of CCK-8 assay and flow cytometry consistently demonstrated that TXL could increase the proportion of viable cells after H/R in a dose-dependent manner, we still could not exclude the possibility that it was the proliferation of HCMs that accounted for the increased proportion of viable HCMs, for activation of p70s6k1 was previously reported to facilitate cellular proliferation (Fenton and Gout, 2011; Xu et al., 2012). However, it was unlikely that TXL could promote the proliferation of HCMs in the serum/glucose-free medium during H/R. In other words, almost no nutrients were available for HCMs to utilize and then undertake proliferation in our experiments simulating I/R. To confirm this speculation, the EdU incorporation assay was used to assess the effects of TXL on the proliferation of HCMs during H/R. EdU is a nucleoside analog of thymidine and can be incorporated into DNA during active DNA synthesis (Salic and Mitchison, 2008), which means that the EdU^+^ cells can be regarded as the newborn cells. Just as predicted, the proportions of replicating cells were lower (~4%) in all groups treated with H/R, compared with that in the normal group (10.43 ± 0.14%). In addition, no between-group difference was observed in the proportion of newborn cells in our experiments simulating I/R (Figure 4C). However, we found that HCMs cultured in complete medium in normal conditions replicated much more quickly if they were treated with 400 μg/mL TXL (data not shown). Taken together, we were inclined to make a conclusion that TXL could protect the HCMs from H/R-induced injury by upregulating p70s6k1 and increasing p-p70s6k1.

### TXL activated the risk pathway by upregulating p70s6k1 and increasing p-p70s6k1 levels *in vivo*

**Figure 5:**
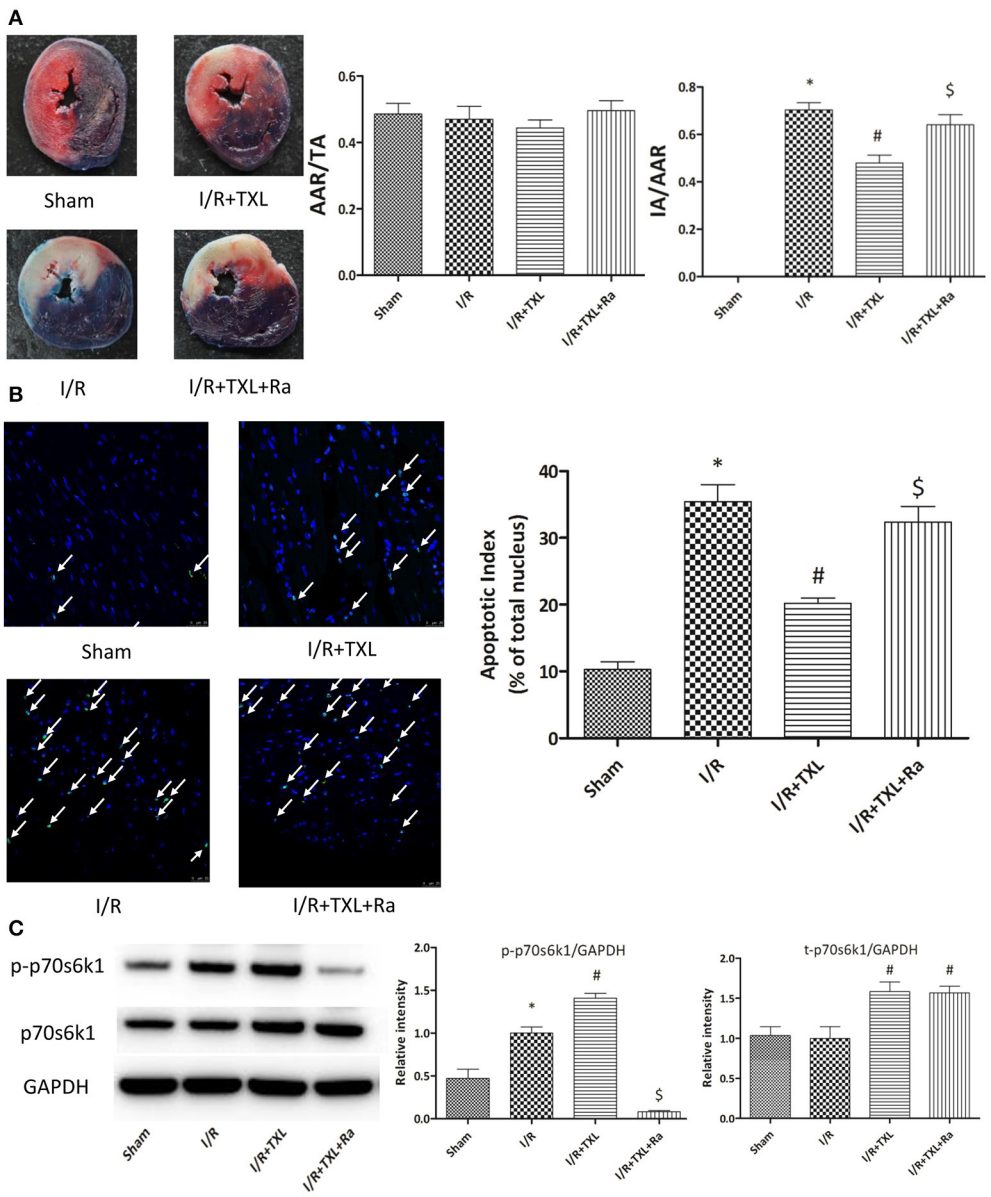
TXL attenuated myocardial reperfusion injury by promoting the expression of p70s6k1 and increasing the phosphorylation of p70s6k1 *in vivo*. **(A)**. Myocardial infraction size assessed by Evans blue/TTC double staining (*n* = 8–9 in each group; rapamycin alone did not affect the infract size after I/R, data not shown). **(B)** The apoptosis of cardiac cells evaluated by TUNEL/DAPI double staining (*n* = 4 in each group TUNEL positive cells are indicated by white arrows). **(C)** The effects of TXL on the protein levels of total p70s6k1 and phosphorylated p70s6k1 in myocardium (*n* = 4 in each group). ^*^P < 0.05 vs. Sham; ^#^*P* < 0.05 vs. I/R; ^$^*P* < 0.05 vs. I/R+TXL; Ra, rapamycin; TA, total cross-sectional heart area; AAR, area at risk; IA, infract area.

TTC is a redox indicator commonly used to indicate cellular respiration. It can be enzymatically reduced to red TPF (1, 3, 5-triphenylformazan) in living tissues by various dehydrogenases (enzymes important in oxidation of organic compounds and thus cellular metabolism), while it remains in its unreacted state in necrotic areas since these enzymes have either denatured or degraded. After being incubated in TTC solution, viable heart muscle will be stained deep red, while infarcted areas will be dyed pale white. Therefore, TTC staining was utilized in our experiments to assess myocardial infarct size after different kinds of treatments. Consistent with the *in vitro* results, our *in vivo* experiment showed that the myocardial infarct size in the TXL group was significantly smaller than that in the I/R group (47.92 ± 3.36% vs. 70.35 ± 3.00%, respectively, *P* < 0.05; Figure 5A). In addition, TUNEL assay was used to detect apoptotic DNA fragmentation, identifying and quantifying apoptotic cells. This assay relied on the use of terminal deoxynucleotidyl transferase(TdT), an enzyme that catalyzed attachment of deoxynucleotides, tagged with fluorescein, to 3′- hydroxyl termini of DNA double strand breaks. As was expected, the number of positive TUNEL stained cardiomyocytes in the I/R group was greater than that in the Sham group, whereas TXL significantly decreased the number of apoptotic cells, compared with in the I/R group (20.22 ± 0.80% vs. 35.45 ± 2.53%, respectively, *P* < 0.05; Figure 5B). These protective effects were partly abrogated by rapamycin (Figures 5A,B).

### TXL downregulated miR-128-3p, a microrna targeting p70s6k1

**Figure 6:**
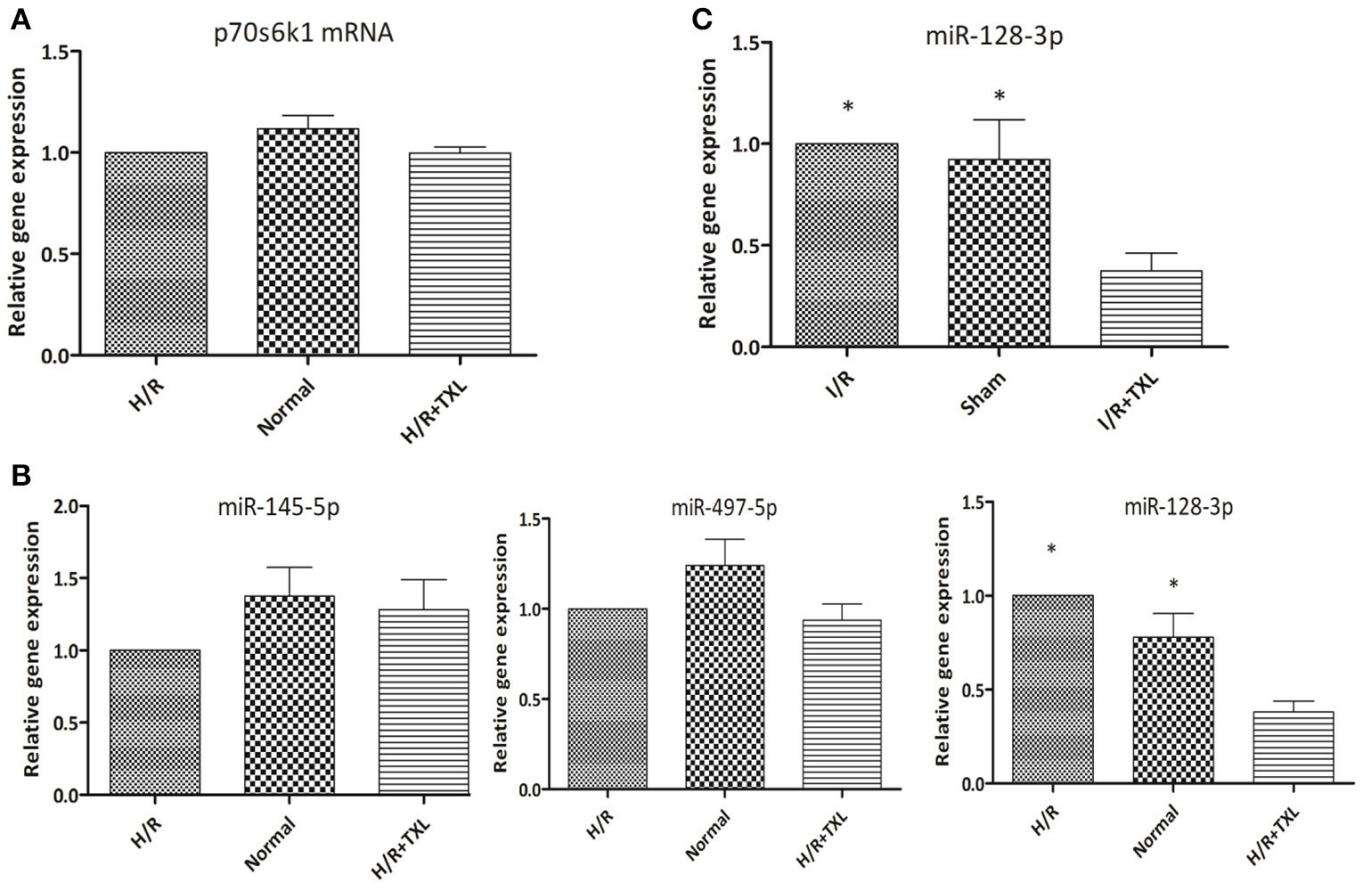
TXL downregulated the level of miR-128-3p in cardiomycytes during H/R or I/R. **(A)** The effect of TXL on the expression of the p70s6k1 mRNA (*n* = 6 in each group). **(B)** The effects of TXL on the expression of microRNAs targeting the mRNA of p70s6k1 in HCMs (*n* = 4 in each group). **(C)** TXL lowered the level of miR-128-3p in rat myocardium (*n* = 4 in each group). ^*^P < 0.05 vs. H/R (I/R)+TXL.

To examine the mechanism of TXL upregulation of p70s6k1 expression, we first assessed the levels of p70s6k1 mRNA in HCMs using real-time PCR. As shown in Figure 6A, TXL did not change p70s6k1 mRNA levels, indicating that it might regulate p70s6k1 at the protein level by promoting translation of p70s6k1 mRNA or inhibiting degradation of p70s6k1 protein.

MicroRNAs are a type of noncoding RNA that regulates gene expression and there are microRNAs that recognize over 60% of human protein-coding genes (Friedman et al., 2009). One mechanism by which microRNAs downregulate gene expression is translational repression, that is, decreasing levels of specific proteins without changing those of their corresponding mRNAs (Bartel, 2004). Because TXL was reported to decrease expression of microRNAs and increase levels of their corresponding proteins under certain conditions (Wang J. Y. et al., 2014; Zhang et al., 2014, 2017), we used quantitative PCR to examine levels of several microRNAs (Shi et al., 2012; Xu et al., 2012, 2015) (miR-497-5p, miR-145-5p, and miR-128-3p) known to target the mRNA of p70s6k1 in HCMs (Figure 6B). Compared with the H/R group, the level of miR-128-3p in HCMs was significantly lower in the TXL treated group (0.38 ± 0.06 vs. 1.00 ± 0.00 in H/R group, *P* < 0.05). There were no significant differences in levels of the other two microRNAs, miR-497-5p and miR-145-5p, in these two groups. Furthermore, our *in vivo* findings confirmed that, compared with that in the I/R group, TXL significantly decreased miR-128-3p levels (0.38 ± 0.09 vs. 1.00 ± 0.00 in the I/R group, *P* < 0.05; Figure 6C) in the rat myocardium after I/R.

### Mir-128-3p was involved in protection by TXL against HCM apoptosis

**Figure 7:**
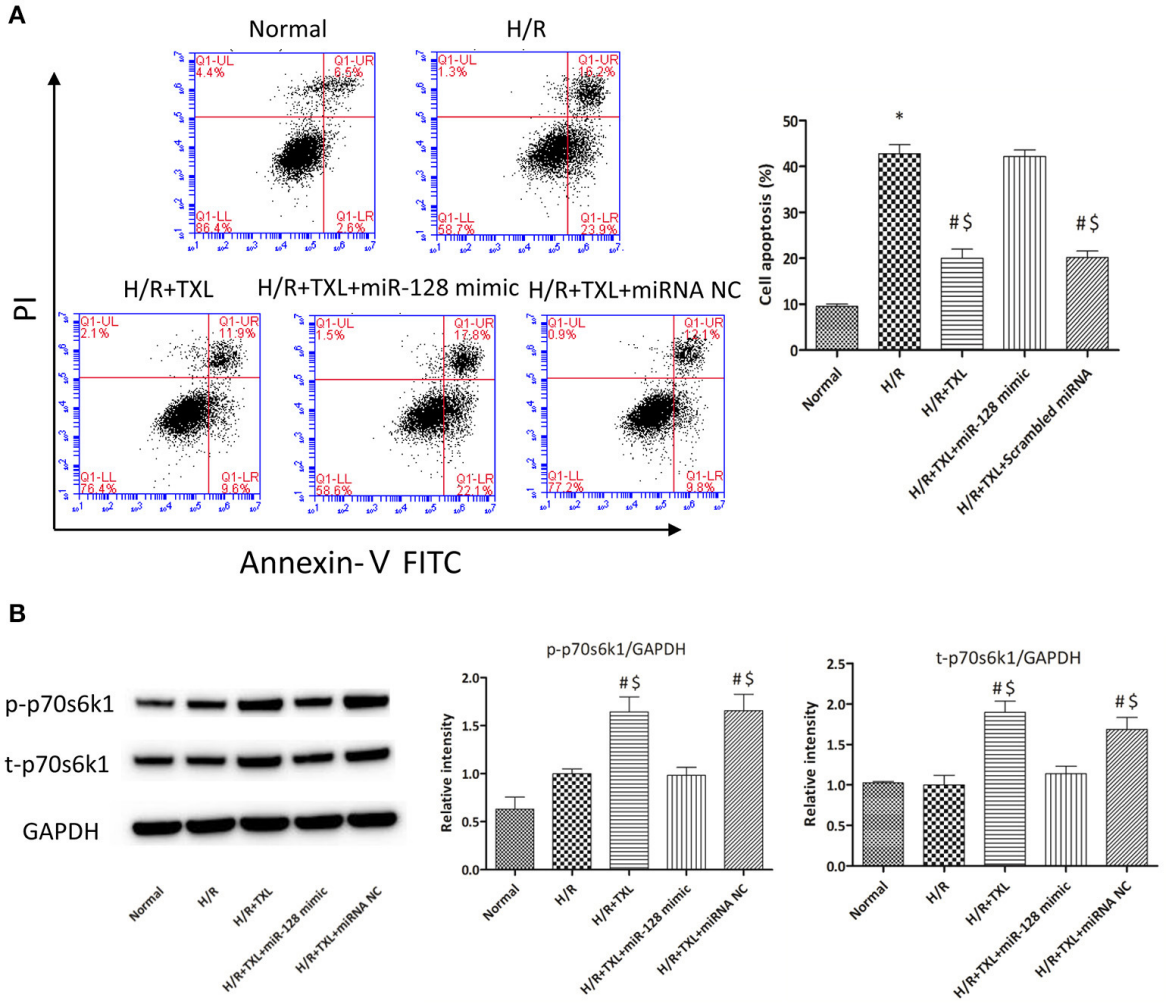
Transfection of miR-128-3p mimic abrogated the anti-apoptotic effects of TXL on HCMs during H/R. **(A)** Apoptotic rates that were determined using flow cytometry (*n* = 3 in each group; miR-128-3p mimic alone did not increase the death of HCMs during H/R, data not shown). **(B)** Western blot analysis of p70s6k1 and its phosphorylated form (*n* = 4 in each group). ^*^P < 0.05 vs. Normal; ^#^*P* < 0.05 vs. H/R; ^$^*P* < 0.05 vs. H/R+TXL+miR-128 mimic; NC, negative control.

To explore whether miR-128-3p was involved in the beneficial effects of TXL against H/R-induced apoptosis, mimics were utilized to upregulate levels of miR-128-3p in HCMs. HCMs were transfected with miR-128-3p mimics, preconditioned with 400 μg/mL TXL and then subjected to H/R. By western blotting analysis, the miR-128-3p mimics downregulated expression and inhibited phosphorylation of p70s6k1 (Figure 7B). Because of the decrease in p-p70s6k1/p70s6k1 levels in HCMs during H/R, TXL pretreatment no longer inhibited cell death in HCMs (Figure 7A), suggesting that upregulation of miR-128-3p abrogated the protective effects of TXL on HCMs.

## Discussion

Our study demonstrated, for the first time, that TXL directly protected cardiomyocytes from H/R injury and, thus, alleviated MIRI. This protective effect was dependent on activation of the RISK pathway, mediated by increased expression and phosphorylation of p70s6k1, rather than by affecting its upstream proteins (Akt and Erk). Furthermore, p70s6k1 upregulation by TXL was attributable to downregulation of miR-128-3p in cardiomyocytes during I/R. We believe that ours is the first study demonstrating that p70s6k1 overexpression in cardiomyocytes was sufficient to reduce MIRI, elucidating potential new strategies to decrease MIRI.

TXL is a multifunctional traditional Chinese medicine reported to exert pleiotropic effects such as anti-fibrosis (Chen W. et al., 2008; Bai et al., 2013; Wang X. et al., 2016), anti-inflammation (Wang et al., 2015), anti-atherogenesis (Wu et al., 2015), anti-apoptosis (Cui et al., 2014; Wei et al., 2016) and improved microvascular barrier function (Liu et al., 2013; Li et al., 2015; Qi et al., 2015; Zheng et al., 2015). This multitude of activities can be explained by the variety of active ingredients in TXL (Cheng et al., 2009). Although our prior studies proved that TXL had infarct-sparing effect during I/R in animals, it remained unclear whether TXL could protect human cardiomyocytes from reperfusion injury. As a consequence, for our *in vitro* experiments, we used human cardiomyocytes, which are widely employed in cardiovascular research (Albrecht-Schgoer et al., 2012; Boon et al., 2013; Baker et al., 2015; Kuo et al., 2015; Nehra et al., 2015; Sharma et al., 2015), rather than cardiomyocytes isolated from animals.

P70s6k is one kinase belonging to the AGC family (Pearce et al., 2010; Prêtre and Wicki, 2017) and can be regulated by the mammalian target of rapamycin (mTOR) pathway (Pearce et al., 2010; Fenton and Gout, 2011; Prêtre and Wicki, 2017). P70s6k was reported to play important roles in diverse cellular processes, including protein synthesis, mRNA processing, glucose homeostasis, cell growth and survival (Fenton and Gout, 2011). Prior studies demonstrated that p70s6k was a protein in the RISK pathway and could be activated by RISK associated kinases like Erk and Akt (Hausenloy and Yellon, 2004; Heusch, 2015). There are two p70S6K subtypes, type 1 (p70S6K1) and type 2 (p70S6K2) (Shima et al., 1998; Tseng et al., 2005), with type 2 barely detectable in the adult heart (Tseng et al., 2005). Given that TXL was shown by several investigators to stimulate cells to secrete VEGF (Wang et al., 2008; Hu et al., 2011; Cui et al., 2016) and that increased p70s6k1 phosphorylation stimulated VEGF expression (Skinner et al., 2004; Fang et al., 2005; Zhou et al., 2007), we examined whether p70s6k1 mediated the protective effects of TXL. Indeed, TXL treatment increased p70s6k1 phosphorylation and, thus, protected cardiomyocytes from H/R-induced injury, by promoting p70s6k1 expression. Pharmacological blockade of p70s6k1 activation with its inhibitor (rapamycin) or siRNA abrogated the beneficial effects of TXL on cardiomyocytes, indicating that TXL protected cardiomyocytes by a pathway involving p70s6k1. This finding, to some extent, was consistent with our previous observations that TXL decreased myocardial infarct size induced by I/R through the protein kinase A (PKA)/eNOS pathway (Cheng et al., 2009; Li et al., 2010; Li X. D. et al., 2013). It was demonstrated that p70s6k activation by neuropeptide Y or cysteine-rich, angiogenic inducer 61, promoted eNOS phosphorylation in endothelial cells and led to blood vessel relaxation (Cheng et al., 2012; Hwang et al., 2015). Moreover, two other studies showed that increased p70s6k1 phosphorylation facilitated PKA expression and enhanced its activity in tissues (Soulard et al., 2010; Jiang et al., 2016). Taken together, it is very likely that TXL can reduce MIRI via the p70s6k1/PKA/eNOS pathway, but further studies will be needed to confirm this speculation. To our knowledge, no practical method of stimulating p70s6k1 phosphorylation, by facilitating p70s6k1 expression, has been developed and translated to the clinic to reduce MIRI. Our study showed, for the first time, that TXL can activate p70s6k1 and alleviate MIRI in this manner.

MicroRNAs, a family of small non-coding single-stranded RNAs, are emerging as robust players regulating genes at the post-transcriptional level. MicroRNAs modulate gene expression by two mechanisms. One is by clearing away mRNA that has sufficient complementarity to the microRNA. The other, when the mRNA does not have sufficient complementarity to be degraded but does have a site complementary to the microRNA, is repressing productive translation (Bartel, 2004). The effects of microRNAs on MIRI depend on their types, because some are protective while others are detrimental (Fan and Yang, 2015). Regulation of microRNAs (inhibition of detrimental microRNAs or overexpression of the protective ones) in I/R has been considered as one novel potential strategy for alleviating MIRI (Hausenloy et al., 2017). Our study demonstrated that TXL promoted p70s6k1 expression without changing p70s6k1 mRNA levels, leading us to investigate the role of microRNAs in the anti-apoptotic effects of TXL. Among the three microRNAs (Shi et al., 2012; Xu et al., 2012, 2015) (miR-497-5p, miR-145-5p, miR-128-3p) previously reported to target p70s6k1 mRNA and inhibit its translation, only miR-128-3p was downregulated by TXL during I/R. MiR-128-3p was implicated as important in multiple physiological and pathophysiological processes, such as angiogenesis, neuronal plasticity, cholesterol metabolism and differentiation (Li M. et al., 2013; Adlakha and Saini, 2014). Moreover, miR-128-3p inhibition in cells enhanced their resistance to detrimental stimuli like chemotherapeutic agents and H/R (Zhu et al., 2011; Chen et al., 2016; Zeng et al., 2016). The protective effects of TXL on HCMs during H/R were largely abolished by transfection with miR-128-3p, indicating that miR-128-3p mediated the beneficial effects of TXL on HCMs during H/R.

The importance of the RISK pathway in cardioprotection was demonstrated by many studies and activation of this pathway is generally associated with increased phosphorylation of Akt or Erk, as well as of their common downstream kinases such as p70s6k1 (Hausenloy and Yellon, 2004; Bouhidel et al., 2008; Heusch, 2015). However, in our study we found that, with inhibition of miR-128-3p, TXL enhanced p70s6k1 phosphorylation and, thus, activated the RISK pathway by facilitating p70s6k1 expression, rather than by activating Akt or Erk. In contrast, we previously showed that TXL upregulated Erk phosphorylation and, subsequently, decreased apoptosis in human cardiac microvascular endothelial cells during H/R (Cui et al., 2014). Discrepancies between these findings may be attributable to differences in responses of endothelial cells and cardiomyocytes to the same stimulus. For example, inhibition of autophagy was reported to protect both primary cardiomyocytes and cardiomyoblasts (Valentim et al., 2006; Zhang et al., 2012) from H/R induced-injury, while it decreased viability of endothelial cells during H/R in our previous study (Cui et al., 2014). In addition, endothelial cells were more vulnerable to I/R, during which apoptosis of endothelial cells preceded that of cardiomyocytes (Scarabelli et al., 2001). Regarding the effects of TXL on Akt during I/R, Yu et al. previously reported that oral administration of TXL (three times a day for 3 days) alleviated cerebral ischemia and reperfusion injury in rats, through Akt activation (Yu et al., 2016). The inconsistencies between these findings and ours probably related to use of different modes of TXL administration. In other studies, TXL could have promoted Akt phosphorylation by upregulating VEGF (Wang B. et al., 2014), the secretion of which can be facilitated by p70s6k1 phosphorylation (Skinner et al., 2004; Fang et al., 2005; Zhou et al., 2007). However, in our study, the single dose given shortly before H/R or IR may not have been sufficient to affect Akt through the p70s6k1/VEGF pathway.

Interestingly, we observed that I/R (or H/R) itself could promote the phosphorylation of p70s6k1, which was consistent with results from other research groups (Chen H. T. et al., 2008; Musiolik et al., 2010; Vilahur et al., 2013). However, it seemed that such upregulation of p70s6k1 could not confer protection on the reperfused-hearts, for rapamycin alone, with the effective inhibition of p70s6k1, did not aggravate MIRI in the current study and others' (Kis et al., 2003b; Pagel et al., 2007; Raphael et al., 2008; Wagner et al., 2010). Moreover, two other research groups demonstrated that rapamycin alone could even alleviate MIRI. For instance, Yang et al. (Yang et al., 2010; Liu et al., 2011) reported that rapamycin could dose-dependently reduce infarct size in the isolated rat hearts if the hearts were perfused with rapamycin for 10 min before I/R. In addition, Kukreja et al. (Khan et al., 2006; Das et al., 2012) proved that whether the mouse hearts were subjected to *in-vivo* I/R or global I/R in Langendorff mode, administration of rapamycin had infarct-sparing effects. The inconsistencies between the findings of these two groups and ours may relate to the I/R modes (isolated hearts or *in vivo* hearts) and species differences. To our knowledge, there may be two reasons explaining the phenomenon that the I/R-induced increase of p-p70s6k1 was not cardioprotective in our study. To begin with, the activation of p70s6k1 by I/R may be too late to reduce MIRI. As previous studies have shown that H_2_O_2_ pretreatment could increase the phosphorylation of p70s6k1 in cells (Tu et al., 2002; Gutierrez-Uzquiza et al., 2012; Huang et al., 2015), the upregulation of p-p70s6k1 after I/R may be attributable to the excessive reactive oxygen species induced by I/R. In this case, the oxidative stress in cardiomyocytes or cardiac issues preceded the increase of p-p70s6k1. As reactive oxygen species had extremely short half-life (Dickinson and Chang, 2011; Das and Roychoudhury, 2014) and damaged cells and issues very rapidly, the activation of p70s6k1 would fail to mitigate the irreversible injuries that had already been induced by the reactive oxygen species. Another explanation is that the amount of p-p70s6k1 increased in I/R was not enough to induce cardioprotection during I/R. Consequently and theoretically, timely and sufficient increase of p-p70s6k1 may have the potential to reduce MIRI. Such a speculation has been preliminarily confirmed by a previous study, in which Zeng et al. demonstrated the cardioprotective effect of sevoflurane postconditioning on MIRI was related to the further activation of p70s6k (Chen H. T. et al., 2008). And our present study directly proved that further enhancement of the phosphorylation of p70s6k1 with TXL could protect the hearts from MIRI.

TXL is a traditional Chinese medicine that was originally approved as an anti-angina drug by the CFDA in 1996. Later, the beneficial effects of its chronic use in other diseases such as hypertension (Wang J. et al., 2014), cardiac ventricle remodeling (Chen W. et al., 2008), diabetes (Chen H. et al., 2008; Zhang et al., 2010) and stroke (Wu et al., 2007) were validated in numerous clinical trials, where severe adverse effects were seldom reported. In other words, TXL is available as a CFDA-approved drug with an acceptable safety profile. Therefore, it is very possible that the acute use of TXL to reduce MIRI will not bring about serious safety problems in patients. As for the efficacy of TXL in attenuating MIRI, the series of studies (Cheng et al., 2009; Li et al., 2010; Li X. D. et al., 2013) from our lab consistently proved that TXL had infarct-sparing effects in small and large animals. Furthermore, the present study demonstrated that TXL exerted a protective effect on human cardiomyocytes during H/R as well, which, to some extent, indicated its efficacy when applied to clinical practice. Taken together, all these findings suggest that TXL has a great potential to emerge as an anti-reperfusion injury therapeutic strategy. However, as the current dosage form of TXL can only be administered orally and then absorbed through the intestinal tract, it takes a relatively long time for TXL to reach its effective blood concentration after oral administration. In order to fully exploit the therapeutic potential of TXL in alleviating MIRI, further studies will be needed to explore how to change the dosage form of TXL and make sure that it can be administrated intravenously.

A major limitation of our study was that we did not identify which ingredients in TXL, alone or in combination, were responsible for activation of the miR-128-3p/p70s6k1 pathway in cardiomyocytes during I/R. Another limitation is that we did not investigate how TXL downregulated miR-128-3p. Further research will be needed to elucidate whether TXL decreases miR-128-3p levels by directly inhibiting the transcription of its gene or, instead, acts in an indirect manner, such as by facilitating expression of endogenous RNAs (e.g., lnc-LAMC2-1:1 Gong et al., 2016) that compete with miR-128-3p.

In conclusion, we reveal for the first time that TXL can directly inhibit cardiomyocyte apoptosis and thus alleviate myocardial reperfusion injury through the miR-128-3p/p70s6k1 pathway. And overexpression of p70s6k1 might represent a new strategy for alleviating myocardial reperfusion injury.

## Author contributions

Designed the experiments: GC, XL, LC, and YY; performed the experiments: GC, CX, JZ, QL, RT, J-yX, XT, PH, and JX; analyzed the data: GC, HC, and CJ; wrote the manuscript: GC and YY; revised the manuscript: GC and YY.

### Conflict of interest statement

The authors declare that the research was conducted in the absence of any commercial or financial relationships that could be construed as a potential conflict of interest.
